# Highly Selective Interfacial Route to Eight-Functional Sucrose Methacrylate for Biocompatible Scaffold Fabrication

**DOI:** 10.3390/polym18121417

**Published:** 2026-06-06

**Authors:** Vladislav Kaplin, Nikolay Glagolev, Nikita Minaev, Evgenii Epifanov, Nadezhda Aksenova, Anastasiia Akovantseva, Tatyana Zarkhina, Olga Vasileva, Elena Kiseleva, Marina Zimens, Anastasia Kuryanova, Gulnaz Mukhametova, Anna Solovieva

**Affiliations:** 1 Semenov Federal Research Center of Chemical Physics, Russian Academy of Sciences, Moscow 119991, Russia; nikgl@mail.ru (N.G.); naksenova@mail.ru (N.A.); akovantseva-a@yandex.ru (A.A.); zarkhina@mail.ru (T.Z.); kiseleva@polymer.chph.ras.ru (E.K.); kuryanovaanastasi@gmail.com (A.K.); marinesko-2@mail.ru (G.M.); ann.solovieva@gmail.com (A.S.); 2 National Research Centre “Kurchatov Institute”, Moscow 123182, Russia; minaevn@gmail.com (N.M.); vivendorigin@yandex.ru (E.E.); 3 Institute for Regenerative Medicine, Sechenov First Moscow State Medical University (Sechenov University), Moscow 119991, Russia; 4 Translational Medicine Research Center, Sirius University of Science and Technology, Sochi 354340, Russia; vasileva.oo@phystech.edu; 5 A.V. Topchiev Institute of Petrochemical Synthesis, Russian Academy of Sciences, Moscow 119991, Russia; zimens@ips.ac.ru; 6 Core Facility Center “Physico-Chemical Studies of New Materials, Substances and Catalytic Systems”, RUDN University (Peoples’ Friendship University of Russia), Moscow 115419, Russia

**Keywords:** sucrose, urethane formation, biopolymer, tissue engineering

## Abstract

The synthesis of reactive sucrose derivatives is of significant interest for the development of novel biocompatible polymers. In this study, an octa-substituted sucrose derivative containing isocyanate groups was synthesized via a urethane-forming reaction carried out in an aprotic solvent at the phase interface. This approach exhibits high selectivity and provides a target product yield of up to 60%. Subsequently, using the same reaction mechanism, the isocyanate derivative was converted into an octa-functional methacrylate derivative capable of forming three-dimensional cross-linked networks. The structures of both the intermediate and final products were confirmed by IR, ^1^H NMR, and mass spectrometry. The sucrose-based prepolymer was further evaluated in the formation of cross-linked structures for potential application as bone-substituting implants. Using various photocuring techniques, including two-photon 3D printing, both plates and microstructured scaffolds were fabricated. These structures exhibited high thermal stability, elastic properties comparable to those of bone tissue, and no toxic effects on cells.

## 1. Introduction

Biodegradable polymers are emerging as promising alternatives to metals and ceramics in regenerative medicine and implantology [1]. This shift is driven by a wide range of unique properties inherent to polymers—features that are often difficult to achieve with other classes of materials [2]. First and foremost, by varying the molecular weight and the composition of polymer functional groups, the properties of polymeric materials can be precisely tailored to meet the requirements of diverse biomedical applications, including bone fixation, structural support, and complete tissue replacement. These modifications also enable adaptation to different tissue types, such as bone, cartilage, ligaments, and skin epithelium, as well as modulation of material–body interactions, including drug release, cell differentiation, and substance diffusion [3,4,5,6,7,8,9,10]. This is made possible by the continually expanding range of biocompatible polymers, advances in their chemical modification and derivative synthesis, as well as the development of physical techniques for processing and structuring final products [11,12]. The principal methods for fabricating implants include injection molding, extrusion, and additive manufacturing, particularly 3D printing [13]. Among these approaches, additive manufacturing holds the greatest promise, as printing parameters directly influence key material properties, including biodegradation rate, stiffness, swelling behavior, cell adhesion, and the internal architecture of scaffolds [14]. Moreover, 3D printing, particularly when based on photocuring techniques, enables the fabrication of implants with exceptionally high (micron-scale) resolution. This level of precision is critical for accurately matching the geometry of tissue defects and for reproducing their complex internal structures [15].

In this context, particular attention is paid to natural and synthetic polymers, especially those modified with acrylate and methacrylate groups, owing to their ability to form three-dimensional crosslinked networks. These polymers include polysaccharides, polyesters, polyalcohols, and proteins [16]. Among these, saccharides deserve special emphasis as a highly promising component for the development of biocompatible polymers [17]. Sugars represent an inexpensive, environmentally friendly raw material produced on an industrial scale [18]. The sucrose molecule contains eight active hydroxyl groups and can serve as a multifunctional branching node within a cross-linked polymer network, acting either as a monomer or as a crosslinking agent, thereby enhancing overall biocompatibility [19,20]. However, the chemical modification of sucrose is complicated by the limited availability of suitable solvents capable of simultaneously dissolving hydrophilic sucrose and hydrophobic reagents and catalysts [21]. The solvents most commonly used for this purpose include DMF, DMSO, pyridine, as well as water-containing mixtures and aqueous solutions [22,23,24]. At the same time, these solvents are difficult to remove, which significantly complicates the production process [25,26,27]. Enzymatic synthesis in aqueous media and mixed solvent systems is frequently employed to introduce new functional groups onto sucrose; this approach typically yields mono-substituted derivatives and, less commonly, di-substituted products [28]. The introduction of a single unsaturated bond is sufficient to enable such a derivative to participate in copolymerization reactions [29]. Moreover, in this case the compound typically retains its water solubility—a property widely utilized in the design of polymeric hydrogel-based drug delivery systems [30,31]. Sucrose derivatives bearing multiple polymerizable groups are even more promising, as they enable the formation of three-dimensional cross-linked networks. For example, a bifunctional sucrose derivative was synthesized using a diisocyanate and hydroxyethyl methacrylate and subsequently evaluated as a crosslinking agent for rubber [32]. Jantas et al. used a crosslinking agent based on methacrylated sucrose to prepare biocompatible hydrogels derived from poly(2-hydroxyethyl methacrylate). Using methacryloyl chloride in an emulsion system, the authors achieved partial substitution of the hydroxyl groups, reaching a degree of substitution of 6.5 [33]. Sucrose itself can also form cross-linked networks without the addition of external crosslinking agents, provided that two or more of its hydroxyl groups are substituted with reactive unsaturated bonds. Using this approach, sucrose diacrylates have been employed to synthesize cross-linked hydrogels [34] and aerogels, which exhibit rapid swelling in aqueous media [35]. Consequently, reactive sucrose derivatives are commonly incorporated into biocompatible polymers and gels, including photo crosslinked materials.

In the area of biocompatible materials, the urethane linkage is a constant companion of hydroxyl-containing compounds [36]. Polyurethanes and urethane-based polymers have long been recognized for their safety and biocompatibility, making them some of the most widely used synthetic biopolymers [37]. Currently, extensive research is focused on urethane polymers and prepolymers processed through various 3D printing techniques (including SLA photopolymerization) for the production of implants with specific geometries and properties [38]. Branched oligomers based on fully substituted sucrose include epoxidized and methacrylated soyates, which are addition products of fatty acids derived from soybean oil [39]. These oligomers were used to successfully prepare durable coatings [40] and were later crosslinked via SLA printing. Previously, we synthesized a photoactive polylactide via a urethane-forming reaction carried out in a supercritical carbon dioxide medium [41]. The material was confirmed to be non-toxic and demonstrated potential as a scaffold base for tissue engineering. In this study, a urethane formation reaction was used to synthesize a multifunctional methacrylate derivative of sucrose—fully substituted sucrose octamethacrylate urethane (OMUS), capable of forming a three-dimensional cross-linked network. This prepolymer can serve as a foundation for biodegradable scaffolds fabricated using photopolymerization-based techniques. Furthermore, CH_2_Cl_2_ was utilized for the first time as a solvent in the synthesis of a sucrose derivative.

## 2. Materials and Methods

The following reagents were used without preliminary purification: sucrose (99%, Central Drug House, Delhi, India), 3-isocyanatomethyl-3,5,5-trimethylcyclohexyl isocyanate (IPDI, 98%, a mixture of isomers, Aldrich, St. Louis, MO, USA), ethylene glycol monomethacrylate (EGM, Aldrich, St. Louis, MO, USA), dibutyltin dilaurate (DBTDL, Aldrich, St. Louis, MO, USA), dichloromethane (HIMFARM, Moscow, Russia, TU 2631-019-44493179-98), and hexane (HIMFARM, Moscow, Russia, TU 2631-158-44493179-13).

### 2.1. Synthesis of a Methacrylate Derivative of Sucrose (OMUS)

Crystalline sucrose was milled in a ball mill (Pulverisette 23, Fritsch, Germany) for 5 min to obtain a homogeneous powder. To synthesize the intermediate isocyanate derivative, 500 mg of sucrose, 2.6 g of IPDI (corresponding to a molar ratio of 1:1 relative to the hydroxyl groups of sucrose), and 300 µL of catalyst were introduced into a sealed 50 mL flask containing 5 mL of CH_2_Cl_2_. The reaction mixture was stirred using a magnetic stirrer at 40 °C for 20 h. Upon completion of the reaction, the insoluble fraction (unreacted sucrose) was removed by centrifugation (Eppendorf 5810, Hamburg, Germany). The resulting isocyanate derivative was then purified by reprecipitation, performed twice by the dropwise addition of the dichloromethane reaction solution into a tenfold excess of hexane. The product was subsequently dried under vacuum at room temperature for 24 h. For the synthesis of the methacrylate derivative, 1.8 g of the isocyanate derivative, 1.5 g of EGM (corresponding to a 1.7-fold molar excess relative to the isocyanate groups of the first-stage product), and 300 µL of catalyst were dissolved in dichloromethane and maintained under identical conditions. The resulting product was purified by reprecipitation in hexane. This procedure was repeated multiple times until the double-bond content (determined by ozonolysis) stabilized, indicating complete removal of unreacted EGM.

### 2.2. Physicochemical Characterization of Reaction Products

Infrared spectra of the starting reagents and the resulting systems were recorded with a Spectrum Two FT-IR Spectrometer (PerkinElmer) operating in Attenuated Total Reflectance mode. The equipment utilized a high-efficiency LiTaO_3_ MIR detector at room temperature and a standard optical setup with KBr windows. Data acquisition covered the 4000–350 cm^−1^ wavenumber range with a resolution of 0.5 cm^−1^. Following initial collection in ATR mode, all spectral data were transformed into IR transmittance format for further evaluation.

Molecular mass was determined using the MALDI-TOF method (Autof MS2600, Autobio Labtec Instruments Co., Zhengzhou, China), employing α-cyano-4-hydroxycinnamic acid as the matrix.

The structures of the octo-isocyanate intermediate and the octo-methacrylate product were confirmed by ^1^H NMR spectroscopy (CDCl_3_, 25 °C, 600 MHz, Bruker Avance 600 spectrometer, Billerica, MA, USA).

Sucrose isocyanate derivative: ^1^H NMR (CDCl_3_, 600 MHz): δ 0.98–1.81 (m, 15H, CH_3_ a, b, c), 3.05–4.00 (m, 3H, CH_2_ d, CH e), 4.00–6.00 (m, 11H, CH_2_, CH protons of sucrose core).

Sucrose methacrylate derivative: ^1^H NMR (CDCl_3_, 600 MHz): δ 0.95–1.81 (m, 15H, CH_3_ a, b, c), 1.96 (m, 3H, CH_3_ h), 2.93–3.9 (m, 3H, CH_2_ d, CH e), 4.33 (d, 4H, CH_2_ i, j), 5.61 (s, 1H, CH_2_ g), 6.15 (s, 1H, CH_2_ f).

The double-bond content was determined using the ozonization method with an ADS-5 instrument, developed and patented at the N.N. Semenov Federal Research Center for Chemical Physics RAS [42,43]. The operating principle of ADS-5 is based on the reaction of ozone with double bonds in an equimolar (1:1) stoichiometry. A weighed portion of the dry sample was dissolved in chloroform, the sample solution was introduced into an ADS-5 reactor, through which an ozone-oxygen mixture was bubbled. The amount of ozone reacted with the double bonds in the sample solution was measured spectrophotometrically by comparing the gas mixture at the reactor outlet with a blank sample. The concentration of double bonds in the solute is equivalent to the amount of absorbed ozone.

### 2.3. Preparation and Investigation of Crosslinked Structures

The cross-linked structures were fabricated in the form of convex plates of 1 cm in diameter and up to 1 mm thick, trapezoids with dimensions of 1 mm × 1 mm × 2 mm, obtained by casting in glass molds, as well as three-dimensional structures referred to as scaffolds. The photoactive composition was a 40% OMUS solution in methylene chloride and a photoinitiator 4,4′-bis(diethylamino)benzophenone (Ethyl Michler ketone, Aldrich, St. Louis, MO, USA), 0.5 wt.% with respect to the weight of the oligomer). To obtain solid hardened structures, the photoactive composition (a viscous liquid) was deposited onto transparent Teflon substrates for plates or into a glass mold for trapezoids. The samples were then irradiated using a light-emitting diode (LED) matrix (Epileds Technologies Inc., Tainan, Taiwan; 50 W, λ = 365 nm, I = 5 W/cm^2^) with an exposure time of 300 s from both sides under out-of-focus light.

Microstructured scaffolds were fabricated within the bulk of the photoactive composition using two-photon polymerization [44,45]. For this purpose, a droplet of approximately 150 µL was deposited onto a coverslip and allowed to air-dry for 24 h. The resulting 5 mm scaffolds consisted of several stacked layers of hexagonally arranged cylinders, with an outer diameter of 250 µm, an inner diameter of 180 µm, and a height of 100 µm. The scaffolds were fabricated using an M3D laser system (LaserNanoFab, Garbsen, Germany) equipped with a submicron positioning stage (Aerotech ABL1000) and a galvanometer scanner (ScanLab HurrySCAN II 14). The radiation source was a femtosecond laser (Avesta Project) operating at a wavelength of λ = 525 nm, with a pulse duration of 200 fs, a repetition rate of 70 MHz, and modulation by acousto-optic modulator up to 1 MHz. A 20× microscope objective with a numerical aperture of 0.4 and a working field diameter of 250 µm was used. Three-dimensional structures were fabricated layer by layer by filling each layer with individual laser “lines” spaced 1 µm apart, with an interlayer spacing of 4 µm. The scanning speed of the focused laser beam was 25 mm/s. The optimal average laser power was determined experimentally in preliminary tests and set to 40 mW. The fabrication time for a single sample with a diameter of approximately 6 mm was about 120 min. Following photocuring, the samples were soaked in THF for several days to remove any uncrosslinked prepolymer.

Differential thermal analysis was performed on crosslinked sucrose derivative (2–5 mg sample mass) using an STA 449 F3 analyzer (NETZCH, Selb, Germany) in air (30 mL/min, 10 °C/min heating rate, relative error ±1.5 °C). The study analyzed weight loss (thermogravimetry, TG, %, accuracy up to 10^−3^ mg) and maximum destruction rate (differential thermogravimetry, DTG, %/min) to evaluate thermo-oxidative stability.

The local Young’s moduli of the surface of crosslinked samples were investigated with the AFM method. Data were collected using a BioScope Resolve microscope (Bruker, Billerica, MA, USA) integrated with an Axio Observer inverted optical microscope (Carl Zeiss, Oberkochen, Germany). Measurements were performed in air using RTESPA-300 rectangular silicon cantilevers (Bruker, Billerica, MA, USA). The actual pre-calibrated values of the spring constant and the tip radius were k = 33.2 N/m and TR = 18 nm. Maps of the surface local Young’s modulus distribution were obtained via the force curve method. Areas of 80 × 80 μm were scanned with a resolution of 40 × 40 pixels. The Hertz model was applied to calculate the Young’s modulus.

Compression tests were performed using a Compact Tabletop Testing Machine EZTest EZ-SX (Shimadzu, Kyoto, Japan) equipped with an SM-500N-168 force transducer (capacity: 500 N; measurement accuracy: ±0.5% of the indicated value). The test specimens were rectangular trapezoids with base dimensions of approximately 1 mm × 1 mm and a height of approximately 2 mm, prepared according to the methodology described above. Immediately prior to testing, the specimen faces were ground, and the exact dimensions were measured using a micrometer. Compression was applied at a loading rate of 1 mm/min. The resulting stress–strain curves were corrected against a blank measurement to eliminate the influence of deformation in the grips and the instrument’s cantilever.

SEM images of the surface of crosslinked samples were obtained using a Phenom ProX scanning electron microscope (Thermo Fisher Scientific, Bleiswijk, The Netherlands).

### 2.4. Cytotoxicity Assessment

HEK293T (American Type Culture Collection, Manassas, VA, USA) and HeLa (Russian Type Culture Collection, Saint Petersburg, Russia) cell cultures were used to evaluate cytotoxicity as well-characterized and widely used model systems [46,47]. The cells were cultured in DMEM/F12 medium (Dulbecco’s Modified Eagle’s Medium/Ham’s mixture; PanEco, Moscow, Russia) supplemented with 10% fetal calf serum (NeoFroxx, Einhausen, Germany) and 2 mM L-glutamine (PanEco, Moscow, Russia). Cultivation was carried out in a CO_2_ incubator (Binder, Tuttlingen, Germany) under a humidified atmosphere containing 5% CO_2_ at 37 °C. The cells were seeded at a density of 1 × 10^6^ per 25 cm^2^ culture flask and passaged every 2–3 days.

**Figure 1:**
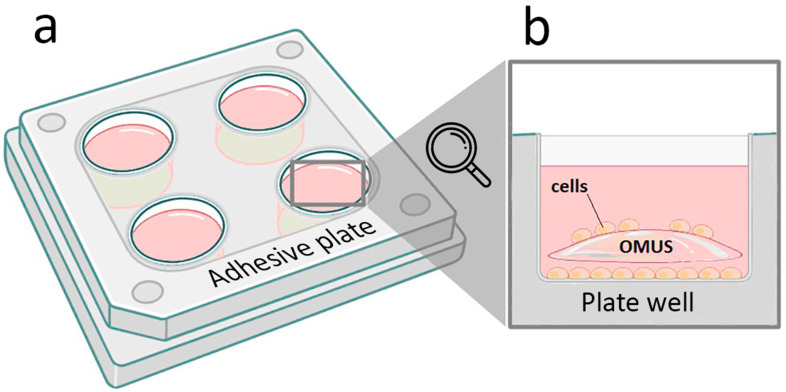
Adhesive plate (**a**) and cell-seeding layout in the singl well (**b**) for the cross-linked sucrose octamethacrylate urethane (OMUS) plate.

One day prior to the experiment, the plates were sterilized by soaking in 95% ethanol. On the day of the experiment, the samples were removed from the ethanol and allowed to dry in a laminar flow hood. Two plates were then placed into each of two 4-well adhesion plates (Nunc, Roskilde, Denmark), leaving two wells empty as controls, and seeded with cells at a density of 30 × 10^3^ cells per well. The volume of medium in the well was 500 µL, and the well surface area was 1.9 cm^2^; the cell seeding layout is presented in Figure 1. After five days, the plates were transferred to PBS, imaged with an inverted phase-contrast microscope (Zeiss, Baden-Württemberg, Germany, Ph 1/0.3) using a 10× objective for visual assessment of adhesion, and then returned to the wells. The cells were subsequently detached following a standard protocol [48]. Dead cells were stained with 0.2% trypan blue (BioLot, Saint-Petersburg, Russia). Live and dead cells were counted using a Countess 3 cell counter (ThermoFisher Scientific, Waltham, MA, USA).

## 3. Results and Discussion

**Scheme 1:**
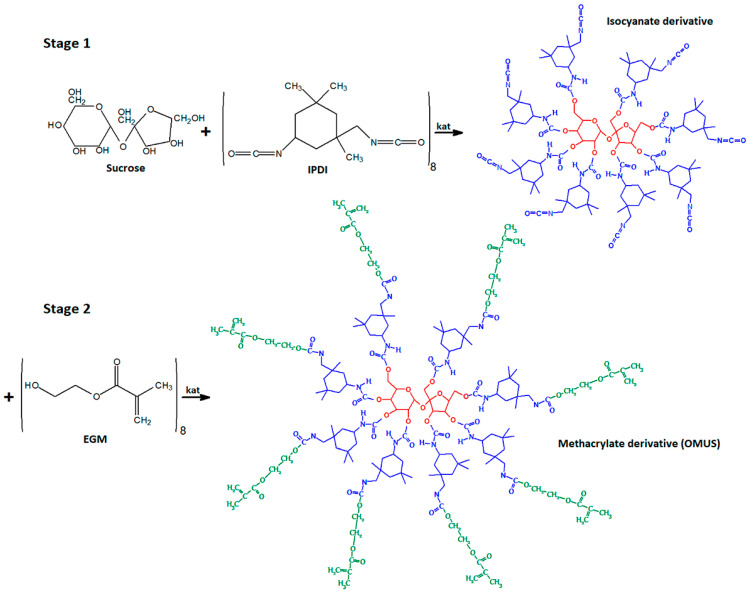
Scheme of the two-stage reaction for grafting methacrylate groups onto sucrose.

Ethylene glycol methacrylate was used as the carrier of reactive methacrylate groups for grafting onto sucrose, with a diisocyanate acting as the linking agent. In this process, the urethane-forming reaction can proceed via all types of hydroxyl groups present in the system (one hydroxyl group from EGM and eight hydroxyl groups from sucrose, exhibiting varying reactivities). In this case, the primary side reaction may involve the interaction of IPDI and EGM in a 1:2 ratio, leading to the formation of urethane dimethacrylate [49]. Therefore, the reaction should be carried out in two stages: in the first stage, sucrose reacts with IPDI to form an isocyanate derivative, which is then reacted with EGM to yield a methacrylate derivative (OMUS) (Scheme 1).

The reactivity of the functional groups in sucrose varies significantly: primary hydroxyl groups are more reactive toward isocyanates than secondary ones [50,51], which leads to difficulties in the synthesis of sucrose derivatives with a functionality greater than three. In addition, as mentioned, complex solvent systems (often containing water) or amphiphilic solvents are typically required. We supposed that such media show a strong affinity for partially substituted sucrose derivatives, which contain both hydroxyl groups and hydrophobic moieties, but a lower affinity for fully substituted derivatives. This is supported by the observation that derivatives with a high degree of substitution were obtained specifically within an emulsion [33]. We therefore hypothesized that aprotic, nonpolar solvents would be more suitable for the synthesis of octa-substituted sucrose derivatives and selected dichloromethane as the reaction medium.

The components of the first-stage reaction mixture (CH_2_Cl_2_, IPDI, DBTDL) are liquid; however, sucrose was found to be insoluble in any of them. In other words, the initial addition reaction of the first diisocyanate group occurs under heterogeneous conditions. As a result, each subsequent addition of an aliphatic IPDI unit increases the solubility of the sucrose derivative, allowing it to transition into the liquid phase, where further addition reactions can proceed at the remaining hydroxyl groups. In the catalytic system used, the cycloaliphatic isocyanate group of IPDI is an order of magnitude more reactive than the aliphatic isocyanate group [52]. Consequently, given the excess of IPDI, resulting from the heterophase nature of the reaction, the addition of free IPDI to hydroxyl groups predominates over side reactions such as dimerization, trimerization, or polyurethane formation.

**Figure 2:**
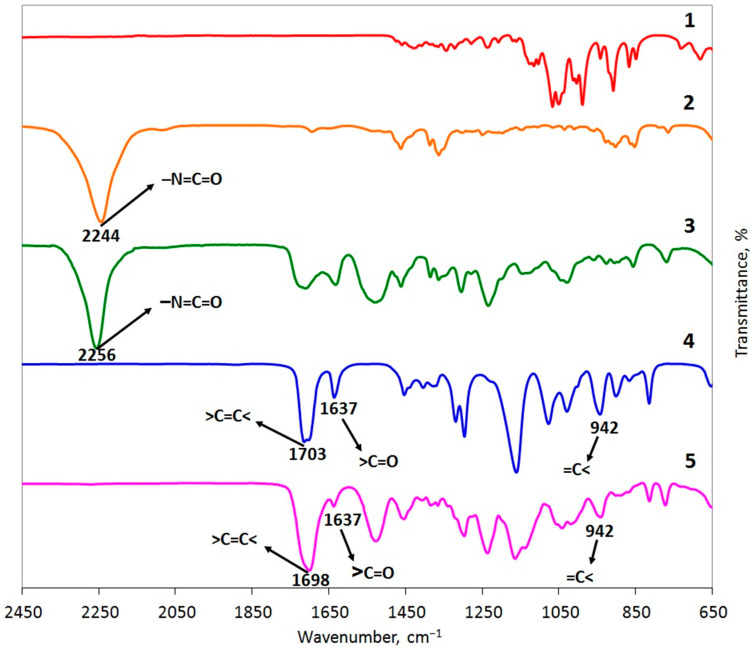
IR spectra of the starting reagents and products: 1—sucrose, 2—IPDI, 3—isocyanate derivative of sucrose, 4—EGM, 5—methacrylate derivative of sucrose.

**Figure 3:**
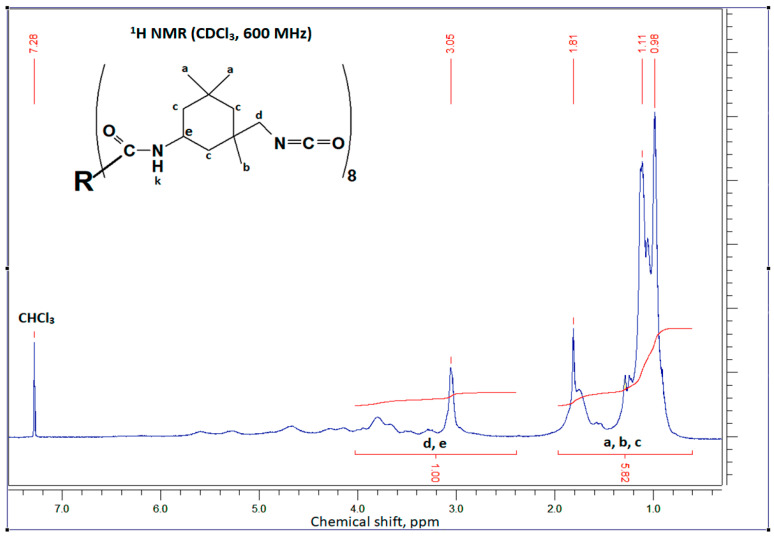
^1^H NMR spectrum of the isocyanate derivative of sucrose. R is a sucrose core.

Figure 2 shows the IR spectra of the starting reagents: sucrose (curve 1), IPDI (curve 2), and EGM (curve 4). The formation of the isocyanate derivative is evidenced by the appearance of an absorption band at 2256 cm^−1^, corresponding to the isocyanate group, in the IR spectrum of the purified intermediate (Figure 2, curve 3). The structure is further confirmed by ^1^H NMR spectroscopy, using sucrose octaacetate as a reference, which is a compound soluble in organic solvents [53]; in this reference, the proton signals of the sucrose core appear in the 4–6 ppm region. Figure 3 presents the ^1^H NMR spectrum of the isocyanate derivative of sucrose, in which signals from both the sucrose core and the protons of the attached IPDI are visible. The signal corresponding to proton “k” originating from the addition of the isocyanate group was expected around 4.7 ppm; however, its low intensity causes it to overlap with the signals of the sucrose core protons. The ^1^H NMR spectra of the starting materials, IPDI and EGM, are provided in Figures S1 and S2.

**Figure 4:**
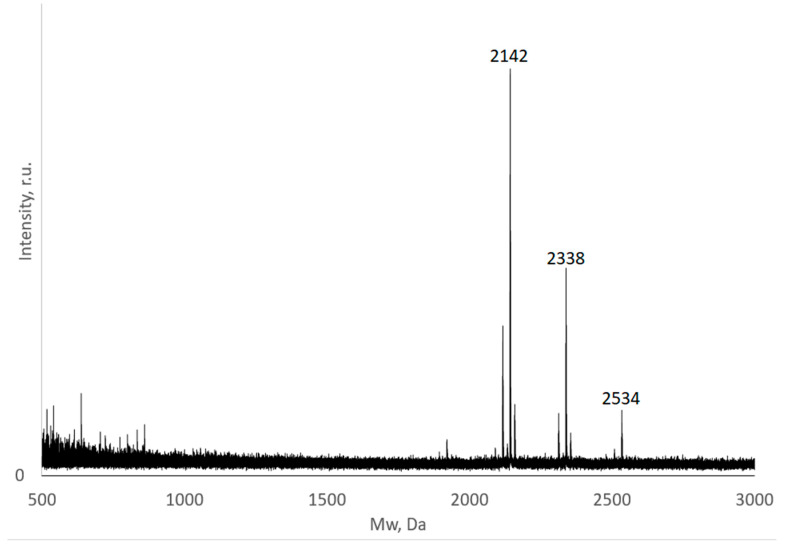
MALDI mass spectrum of the reprecipitated reaction mixture after the first stage.

Reactions involving the functional groups of sucrose frequently yield a mixture of mono-, di-, tri-, and higher-substituted derivatives simultaneously [54]; therefore, controlling selectivity is a primary objective. To determine the degree of substitution of sucrose and the product distribution, mass analysis was performed using the MALDI-TOF technique. Analysis of the intermediate isocyanate derivative of sucrose (purified via reprecipitation) revealed the presence of only a single product: the octa-substituted derivative. In the spectrum (Figure 4), the product is represented by a main peak at 2142 Da, corresponding to a sodium-ionized molecule with a calculated mass of 2120 Da. Two satellite peaks at 2338 Da (+196 Da) and 2534 Da (+392 Da) correspond to octa-substituted sucrose in which one or two additional IPDI have attached to hydroxyl groups, forming a dimer (diurethane) as a result of the interaction of isocyanate groups with residual moisture in the solvent. Notably, no other products corresponding to lower degrees of substitution are observed in the spectrum, while the reaction yield is 61.8 ± 10.7% (determined gravimetrically based on at least 8 experiments, at a significance level of α = 0.05). The resulting isocyanate derivative is highly soluble in organic solvents and EGM; consequently, the subsequent stage proceeds in a homogeneous phase.

**Figure 5:**
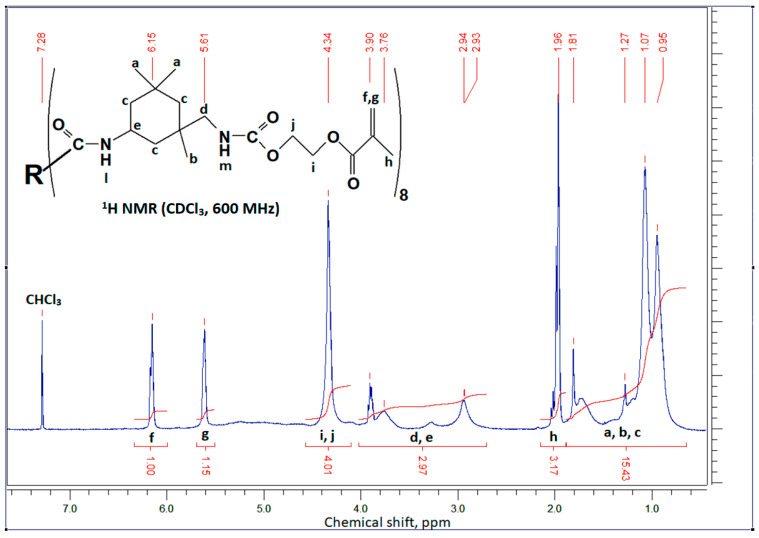
^1^H NMR spectrum of sucrose methacrylate. R is a sucrose core.

**Figure 6:**
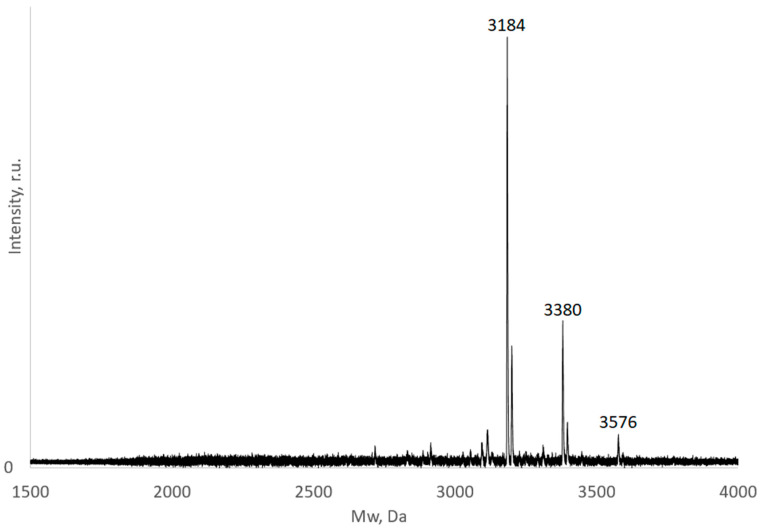
MALDI mass spectrum of the reprecipitated reaction mixture after the second stage.

In the second stage, the intermediate sucrose–IPDI product reacts with EGM; accordingly, in the IR spectrum of the purified product (after removal of residual EGM), the absorption band associated with isocyanate groups disappears, while bands characteristic of carbonyl and acrylate groups appear (Figure 2, curve 5), confirming the formation of the methacrylate derivative. The ^1^H NMR spectrum of the product (Figure 5) displays signals corresponding to protons from both the isocyanate and methacrylate moieties in an integral ratio of 1:1, indicating that the reaction proceeded to completion with respect to the isocyanate groups. However, due to the high intensity of the signals from the aliphatic protons, the signals from the protons of the sucrose core, as well as those from the single protons attached to nitrogen atoms “l” and “m”, are poorly resolved. The mass spectrum of the product from the second stage (Figure 6) shows peaks corresponding to sodium-ionized sucrose methacrylate derivatives at 3184, 3380, and 3576 Da. These masses correspond to the isocyanate derivatives formed in the first stage, now bearing eight terminal EGM fragments (+130.14 Da). Notably, no peaks corresponding to the initial isocyanate derivative are observed, indicating that the second stage of EGM addition proceeds to completion. Consequently, the overall yield is primarily determined by the efficiency of the first stage. The final product also exhibits high solubility in common organic solvents, including acetone, chloroform, and dichloromethane.

The degree of sucrose modification was further confirmed by determining the double bond content in the final product. According to ozonolysis data, the concentration of double bonds in the prepolymer is 2.45 ± 0.35 × 10^−3^ mol/g (at a significance level of α = 0.05), which is close to that for an octa-substituted derivative (2.49 × 10^−3^ mol/g).

**Figure 7:**
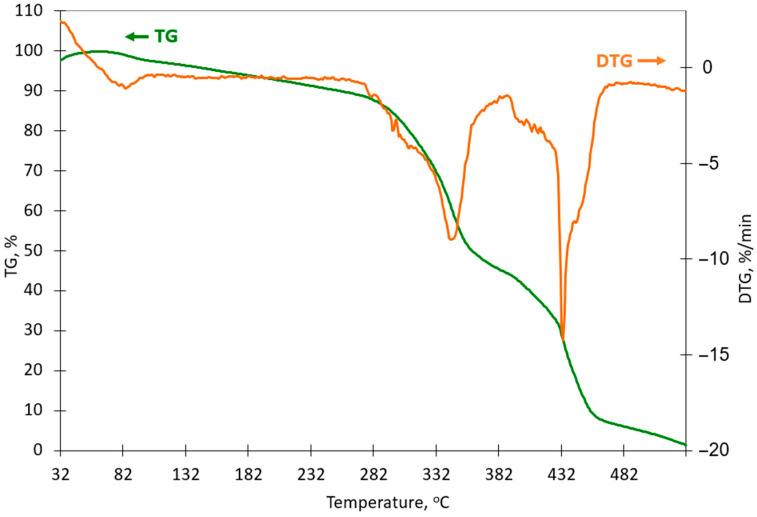
Thermogravimetric (TG) and derivative thermogravimetric (DTG) curves of UV-LED-crosslinked OMUS during heating.

Thermogravimetric analysis indicates that the primary stage of mass loss, which is associated with the onset of thermal degradation of the matrix, begins at approximately 270 °C (Figure 7). The moderate mass loss observed in the initial temperature range (70–270 °C) can likely be attributed to the release of residual solvent from the bulk of the sample. Such thermal stability suggests that the scaffolds can withstand thermal sterilization, for example by autoclaving, prior to implantation [55].

**Figure 8:**
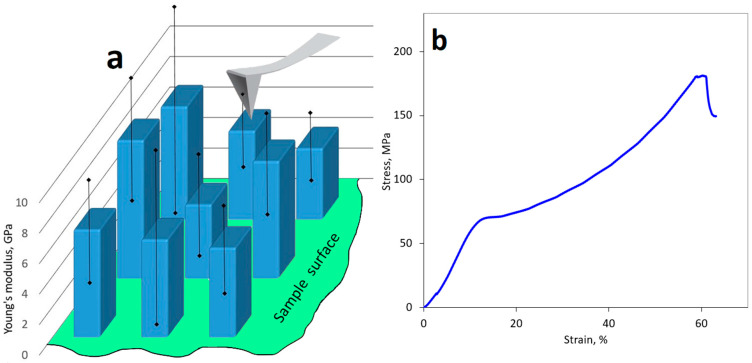
(**a**) Local Young’s modulus values (**a**) averaged within each scan area (map) on the surface of UV-LED cross-linked OMUS. Vertical lines indicate standard deviations. (**b**) Typical stress–strain diagram for a prismatic cross-linked OMUS sample tested in compression.

While SEM micrographs of the UV-LED crosslinked samples (Figures S3 and S4) show a featureless surface, nanoindentation reveals some non-uniformity. Local Young’s modulus values were determined for the LED-cured plates using nanoindentation. The spatial distribution of local Young’s modulus values exhibited a non-uniformity of about 30%, most likely due to inhomogeneous photocrosslinking within the bulk of the material (Figure 8a). The mean value was 6.13 ± 2.9 GPa (at a significance level of α = 0.05). To determine the elastic modulus, the material was subjected to compression testing, a loading mode more representative of the mechanical conditions experienced by bone tissue. Stress–strain curves were obtained for a series of 10 specimens (Figure 8b). The elastic modulus was calculated from the slope of the initial linear region of the curve, corresponding to elastic deformation of the specimen, and was found to be approximately 1.18 ± 0.13 GPa (at a significance level of α = 0.05). This elastic modulus is comparable to that of human cancellous (spongy) bone [56]. According to the stress–strain curves, the material exhibits an ultimate strength of approximately 70 ± 7 MPa (at a significance level of α = 0.05). Upon further loading, irreversible deformation of the cross-linked polymer occurs, followed by specimen failure. It is noteworthy that the material undergoes substantial irreversible deformation—up to 55% strain—without fracture, indicating high static toughness associated with the progressive rupture of cross-links within the polymer network. This can be beneficial in withstanding cyclical loads on the musculoskeletal system [57]. The lower elastic modulus values observed for the bulk specimens, compared to the local elastic modulus measurements, are likely attributable to non-uniform cross-linking throughout the polymer volume, as well as to defects and geometric inaccuracies introduced during fabrication of the trapezoidal specimens. Nevertheless, the mechanical properties of specimens fabricated via photocrosslinking of the synthesized prepolymer can be intentionally adjusted to better match the characteristics of specific regions of bone tissue [58]. During the production process, it is possible to control such factors influencing the final strength of the material as: the number of reactive groups grafted onto the molecule (ranging from 1 to 8), the concentration of the prepolymer and photoinitiator in the photopolymerizable mixture, the duration, energy, and method of irradiation, and the introduction of additional plasticizers or low-molecular-weight crosslinking agents.

**Figure 9:**
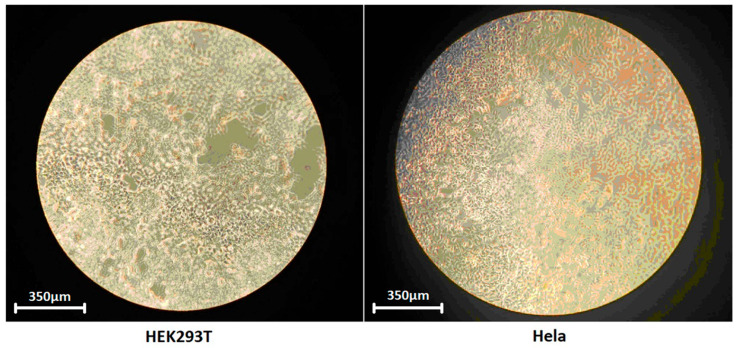
Images of a layer of HEK293T and HeLa cells on cross-linked OMUS plates, transferred into a PBS solution.

Cell studies showed that both HEK293T and HeLa cultures formed a continuous monolayer on the OMUS plate, which remained intact after transfer to a PBS solution (Figure 9), indicating adequate cell adhesion despite the hydrophobic nature of the material. Not all cells in the image are in focus due to the curvature of the film.

The results of cell counting on the test material after 5 days of incubation are presented in Table 1. The viability assessment indicates only a minor decrease in survival (averaging 13% for HEK293T and 7% for HeLa), confirming the absence of cytotoxic effects. However, the number of cells cultured on OMUS plates is approximately half of that observed without the plates, suggesting a moderate cytostatic effect that slows down cell division. This effect may be attributed, among other factors, to the residual presence of unsaturated bonds in methacrylate groups that did not undergo crosslinking under the conditions of bulk polymerization.

**Figure 10:**
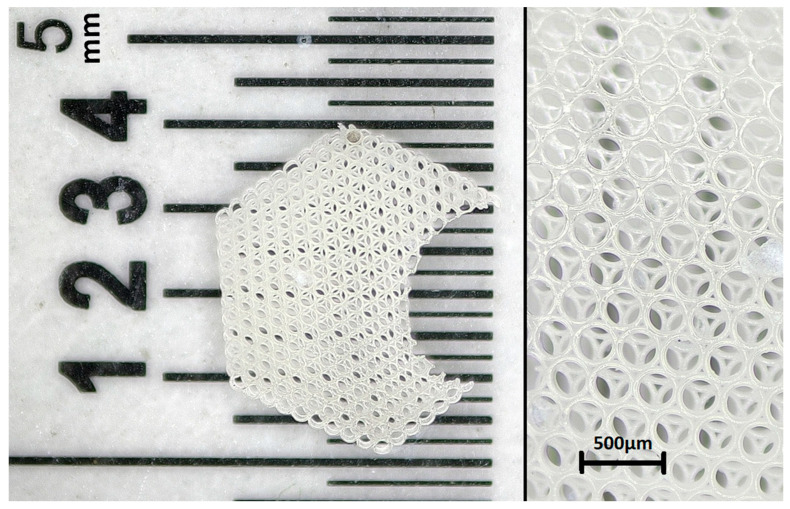
A three-dimensional scaffold, fabricated via two-photon polymerization from OMUS and consisting of two layers of rings with a diameter of 250 µm.

An example of a structure fabricated from methacrylated sucrose via two-photon polymerization is presented in Figure 10. It is evident that the prepolymer supports micrometer-scale printing resolution (ring diameter: 250 µm), while the crosslinked ring walls remain undeformed by heating during the printing process or during washing in an organic solvent. The resulting structure was evaluated for its correspondence to the original three-dimensional model using optical microscopy. Overall, a shrinkage effect of approximately 4–5% was observed, while the shape and contours of the figure were fabricated with an accuracy of several micrometers. This enables the use of photoactive compositions based on OMUS as a material for printing microstructured implants and scaffold carriers for cell cultures.

## 4. Conclusions

An octa-functional methacrylate derivative of sucrose has been synthesized for the first time. A two-step heterophase urethane-forming reaction, carried out using a diisocyanate with non-equivalent isocyanate groups, enabled the synthesis of the target product with a yield of up to 60% and a high degree of selectivity. The octa-substituted structure of the oligomeric sucrose derivative is confirmed by IR, ^1^H NMR, and MALDI spectroscopy. In the presence of initiators, the synthesized compound undergoes polymerization to form a cross-linked polymer, enabling the fabrication of three-dimensional objects (bulk-crosslinked trapezoidal and microstructured scaffolds) using LED-photocuring, photostereolithography and laser microstructuring techniques. It has been demonstrated that the resulting cross-linked structures exhibit high thermal stability (up to 270 °C). Under mechanical testing, the bulk-crosslinked polymer exhibits an elastic modulus of approximately 1 GPa and a tensile strength of up to 70 MPa, which is comparable to the mechanical properties of bone tissue. An irreversible deformation of up to 55% without specimen failure constitutes an advantage under static loading conditions. Local nanoindentation reveals a minor surface non-uniformity yet confirms the order of magnitude of the elastic modulus. The crosslinked scaffolds exhibit no cytotoxic effects on living cells, making the material suitable for bone-replacement implants.

## Figures and Tables

**Table 1:** Cell count data for HEK293T and HeLa cells cultured on cross-linked OMUS plates and in control samples.

	Number of Cells Per Well, ×10^3^	Viability, %
HEK293T blank	450	98
HEK293T blank	408	95
HEK293T + OMUS	200	96
HEK293T + OMUS	190	81
HeLa blank	450	92
HeLa blank	415	95
HeLa + OMUS	296	89
HeLa + OMUS	142	84

## Data Availability

The original contributions presented in this study are included in the article/Supplementary Materials. Further inquiries can be directed to the corresponding authors.

## Supplementary Materials

The following supporting information can be downloaded at https://www.mdpi.com/article/10.3390/polym18121417/s1, Figure S1: ^1^H-NMR spectrum of the initial 3-isocyanatomethyl-3,5,5-trimethylcyclohexyl isocyanate; Figure S2. ^1^H-NMR spectrum of the initial ethyleneglycol monomethacrylate. Figure S3. SEM image of the free surface and fracture surface of the UV-LED crosslinked OMUS. Magnification: ×1200; Figure S4. SEM image of the free surface and fracture surface of the UV-LED crosslinked OMUS. Magnification: ×9500.

## References

[1] Sharma B., Sharma S., Jain P. (2020). Leveraging advances in chemistry to design biodegradable polymeric implants using chitosan and other biomaterials. Int. J. Biol. Macromol..

[2] Oladele I.O., Oki V.O., Omotosho T.F., Adebanjo M.B., Ayanleye O.T., Adekola S.A. (2025). Sustainable polymer and polymer-based composite materials for extreme conditions and demanding applications—A review on pushing boundaries in materials science. Next Mater..

[3] Mei D., You R., Wang L., Ma C., Bai Y., Li X. (2026). Molecular-weight-dependent biodegradation of silk I type silk fibroin scaffolds. Polym. Degrad. Stab..

[4] Farrar D.F., Gillson R.K. (2002). Hydrolytic degradation of polyglyconate B: The relationship between degradation time, strength and molecular weight. Biomaterials.

[5] Niinomi M., Wang L., Enjitsu T., Fukunaga K.-I. (2001). Fatigue characteristics of ultra high molecular weight polyethylene with different molecular weight for implant material. J. Mater. Sci. Mater. Med..

[6] Bodde E.W.H., Habraken W.J.E.M., Mikos A.G., Spauwen P.H.M., Jansen J.A. (2009). Effect of Polymer Molecular Weight on the Bone Biological Activity of Biodegradable Polymer/Calcium Phosphate Cement Composites. Tissue Eng. Part A.

[7] Thevenot P., Hu W., Tang L. (2008). Surface Chemistry Influences Implant Biocompatibility. Curr. Top. Med. Chem..

[8] Profire L., Dragostin O. (2017). Molecular weight of polymers used in biomedical applications. Characterization of Polymeric Biomaterials.

[9] Saha S., Lin X., Zhou L., Xue A., Gosselin E., Chothe P.P., Darji M., Lu X., Yang W. (2025). Evaluation of the impact of the polymer end groups and molecular weight on in vitro and in vivo performances of PLGA based in situ forming implants for ketoprofen. J. Pharm. Sci..

[10] Sunoqrot S., Alsadi A., Tarawneh O., Hamed R. (2017). Polymer type and molecular weight dictate the encapsulation efficiency and release of Quercetin from polymeric micelles. Colloid Polym. Sci..

[11] Kurowiak J., Klekiel T., Bedzinski R. (2023). Biodegradable Polymers in Biomedical Applications: A Review—Developments, Perspectives and Future Challenges. Int. J. Mol. Sci..

[12] Nikhade R., Jaiswal A. (2025). Advancements in Polymer Modification: A Comprehensive Review on Techniques. Int. J. Pharm. Sci. Med..

[13] Kirilova Yeazel T.R., Asheghali D., Petersen S.R., Dort S., Gall K., Becker M.L. (2021). Fabrication of Biomedical Scaffolds Using Biodegradable Polymers. Chem. Rev..

[14] Gide K.M., Islam S., Bagheri Z.S. (2022). Polymer-Based Materials Built with Additive Manufacturing Methods for Orthopedic Applications: A Review. J. Compos. Sci..

[15] Elkhoury K., Zuazola J., Vijayavenkatarraman S. (2023). Bioprinting the future using light: A review on photocrosslinking reactions, photoreactive groups, and photoinitiators. SLAS Technol..

[16] Zhang Q., Bei H.-P., Zhao M., Dong Z., Zhao X. (2022). Shedding light on 3D printing: Printing photo-crosslinkable constructs for tissue engineering. Biomaterials.

[17] Galbis J.A., Garcia-Martin M.D.G., Violante de Paz M., Galbis E. (2016). Synthetic Polymers from Sugar-Based Monomers. Chem. Rev..

[18] Jarosz S., Sokolowska P., Szyszka L. (2020). Synthesis of fine chemicals with high added value from sucrose: Towards sucrose-based macrocycles. Tetrahedron Lett..

[19] Piroonpan T., Booncharoen K., Pasanphan W. (2022). Sugar-Core Synthesized Multibranched Polylactic Acid and Its Diacrylate Blends as a UV LED-Curable Coating with Enhanced Toughness and Performance. ACS Sustain. Chem. Eng..

[20] Nagy L., Nagy M., Vadkerti B., Daroczi L., Deak G., Zsuga M., Keki S. (2019). Designed Polyurethanes for Potential Biomedical and Pharmaceutical Applications: Novel Synthetic Strategy for Preparing Sucrose Containing Biocompatible and Biodegradable Polyurethane Networks. Polymers.

[21] Queneau Y., Fitremann J., Trombotto S. (2004). The chemistry of unprotected sucrose: The selectivity issue. Comptes Rendus Chim..

[22] Queneau B.Y., Jarosz S., Lewandowski B., Fitremann J. (2007). Sucrose Chemistry and Applications of Sucrochemicals. Adv. Carbohydr. Chem. Biochem..

[23] de Gouvea R.S.O., Felisberti M.I. (2021). Stimuli-Responsive Hydrogels Based on Random Copolymers of the Sucrose Methacrylate. Macromol. Mater. Eng..

[24] Sterling J.C.R., Ortega J.O., Metzker G., Boscolo M., Vargas J.A.M. (2022). Fast and selective synthesis of mono-substituted sucrose methacrylate ester monomer. Carbohydr. Res..

[25] Pan C.-T., Ghosh S., Wu C.-M., Shiue Y.-L., Rahim M.S. (2026). Review: Solvent systems in electrospinning—Polymer compatibility, toxicological risks, and environmental considerations. J. Mater. Sci..

[26] Dixit K., Athawale R.B., Singh S. (2015). Quality control of residual solvent content in polymeric microparticles. J. Microencapsul..

[27] Ke K., Wei X.-F., Bao R.-Y., Yang W., Luo Y., Xie B.-H., Yang M.-B. (2014). Contribution of residual solvent to the nucleation and reinforcement of poly (vinylidene fluoride). Polym. Test..

[28] Oliveira H.F.N., Felisberti M.I. (2013). Amphiphilic copolymers of sucrose methacrylate and acrylic monomers: Bio-based materials from renewable resource. Carbohydr. Polym..

[29] Marcilli R.H.M., Petzhold C.L., Felisberti M.I. (2020). Triblock Copolymers Based on Sucrose Methacrylate and Methyl Methacrylate: RAFT Polymerization and Self-Assembly. Macromol. Chem. Phys..

[30] Huang S., Wang L., Shang Q. (2017). Development and evaluation of a novel polymeric hydrogel of sucrose acrylate-co-polymethylacrylic acid for oral curcumin delivery. J. Biomater. Sci. Polym. Ed..

[31] Perin G.B., Fonseca L.P., Felisberti M.I. (2019). Macroporous hydrogels based on carbohydrates monomethacrylates and dimethacrylates: Singular properties from carbohydrate-based crosslinkers. J. Chem. Technol. Biotechnol..

[32] Abdel-Hakim A., EI-Gamal A.A., EL-Zayat M.M., Sadek A.M. (2021). Effect of novel sucrose based polyfunctional monomer on physico-mechanical and electrical properties of irradiated EPDM. Radiat. Phys. Chem..

[33] Jantas R., Herczynska L., Potakowska J. (2011). Preparation and cross-linking properties of methacrylated sucrose. Polym. Bull..

[34] Ortiz R.A., Martinez A.Y.R., Valdez A.E.G., Duarte M.L.B. (2010). Preparation of a crosslinked sucrose polymer by thiol–ene photopolymerization using dithiothreitol as comonomer. Carbohydr. Polym..

[35] Chen J., Park K. (2000). Synthesis of fast-swelling, superporous sucrose hydrogels. Carbohydr. Polym..

[36] Zoltowska K., Piotrowska U., Oledzka E., Kuras M., Zgadzaj A., Sobczak M. (2017). Biodegradable Poly(ester-urethane) Carriers Exhibiting Controlled Release of Epirubicin. Pharm. Res..

[37] Singh S., Paswan K.K., Kumar A., Gupta V., Sonker M., Khan M.A., Kumar A., Shreyash N. (2023). Recent Advancements in Polyurethane-based Tissue Engineering. ACS Appl. Bio Mater..

[38] Griffin M., Castro N., Bas O., Saifzadeh S., Butler P., Dietmar H. (2020). The current versatility of polyurethane 3D-printing for biomedical applications. Tissue Eng..

[39] Silbert S.D., Simpson P., Setien R., Holthaus M., Scala J.J.L., Ulven C., Webster D.C. (2020). Exploration of Bio-based Functionalized Sucrose Ester Resins for Additive Manufacturing via Stereolithography. ACS Appl. Polym. Mater..

[40] Yu A.Z., Sahouani J.M., Webster D.C. (2018). Highly functional methacrylated bio-based resins for UV-curable coatings. Prog. Org. Coat..

[41] Kaplin V.S., Glagolev N.N., Shashkova V.T., Matveeva I.A., Shershnev I.V., Zarkhina T.S., Minaev N.V., Aksenova N.A., Shavkuta B.S., Bezrukov E.A. (2020). Photocurable Methacrylate Derivatives of Polylactide: A Two-Stage Synthesis in Supercritical Carbon Dioxide and 3D Laser Structuring. Polymers.

[42] Brazhnikov E.M., Lisitsyn D.M., Pebalk V.V., Razumovsky S.D., Russiyan E.K., Shalomeev A.S., Chirkov N.M. (1968). A Method for the Quantitative Determination of Double Bonds in Organic Compounds.

[43] Brazhnikov E.M., Gerbich V.I., Krinsky I.V., Lisitsyn D.M., Moskvin V.F., Razumovsky S.D., Russiyan E.K., Trubnikov G.R., Chernykh V.I., Chirkov N.M. (1984). A Device for the Determination of Unsaturation in Organic Compounds.

[44] Demina T.S., Bardakova K.N., Svidchenko E.A., Minaev N.V., Pudovkina G.I., Novikov M.M., Butnaru D.V., Surin N.M., Akopova T.A., Bagratashvili V.N. (2016). Fabrication of Microstructured Materials Based on Chitosan and D,L-Lactide Copolymers Using Laser-Induced Microstereolithography. High Energy Chem..

[45] Timashev P.S., Bardakova K.N., Minaev N.V., Demina T.S., Mishchenko T.A., Miroshina E.V., Akovantseva A.A., Koroleva A.V., Asyutin D.S., Pimenova L.F. (2016). Compatibility of Cells of the Nervous System with Structured Biodegradable Chitosan-Based Hydrogel Matrices. Appl. Biochem. Microbiol..

[46] Jiang X.J., Lu C., Tang M., Yang Z., Jia W., Ma Y., Jia P., Pei D., Wang H. (2018). Nanotoxicity of Silver Nanoparticles on HEK293T Cells: A Combined Study Using Biomechanical and Biological Techniques. ACS Omega.

[47] Liu X., Shan K., Shao X., Shi X., Liu Y.H.Z., Jacob J.A., Deng L. (2021). Nanotoxic Effects of Silver Nanoparticles on Normal HEK-293 Cells in Comparison to Cancerous HeLa Cell Line. Int. J. Nanomed..

[48] Rogovaya O.S., Alpeeva E.V., Ruchko E.S., Eremeev A.V., Vorotelyak E.A. (2023). Survival of Human Cells in Tissue-engineered Constructs Stored at Room Temperature. Bull. Russ. State Med. Univ..

[49] Shashkova V.T., Matveeva I.A., Glagolev N.N., Zarkhina T.S., Timashev P.S., Bagratashvili V.N., Soloveva A.B. (2016). Selective Modification of Polylactide by Introducing Acrylate Groups: IR Spectroscopy, Gel Permeation Chromatography, and Differential Thermal Analysis. Russ. J. Phys. Chem. A.

[50] Nagy L., Vadkerti B., Batta G., Feher P.P., Zsuga M., Keki S. (2019). Eight out of Eight: A Detailed Kinetic Study on the Reactivities of the Eight Hydroxyl groups of Sucrose with Phenyl Isocyanate. New J. Chem..

[51] Nagy L., Nagy T., Kuki A., Purgel M., Zsuga M., Keki S. (2017). Kinetics of Uncatalyzed Reactions of 2,4-and 4,4-Diphenylmethane-Diisocyanate with Primary and Secondary Alcohols. Int. J. Chem. Kinet..

[52] Karpov S.V., Lodygina V.P., Komratova V.V., Dzhalmukhanova A.S., Malkov G.V., Badamshina E.R. (2016). Kinetics of Urethane Formation from Isophorone Diisocyanate: The Catalyst and Solvent Effects. Kinet. Catal..

[53] Bruyn A.D., Anteunis M., Verhegge G. (1975). Sucrose Octa-acetate: The Complete Analysis of the 1H-n.m.r. spectrum. Carbohydr. Res..

[54] Huang D., Jiang X., Zhu H., Fu X., Zhong K., Gao W. (2010). Improved synthesis of sucrose fatty acid monoesters under ultrasonic irradiation. Ultrason. Sonochem..

[55] Beh C.C. (2023). Sterilization Techniques of Biomaterials (Implants and Medical Devices). Biomat. Biopol..

[56] Wu D., Isaksson P., Ferguson S.J., Persson C. (2018). Young’s Modulus of Trabecular Bone at the Tissue Level: A Review. Acta Biomater..

[57] Miller A.T., Safranski D.L., Smith K.E., Guldberg R.E., Gall K. (2016). Compressive cyclic ratcheting and fatigue of synthetic, soft biomedical polymers in solution. J. Mech. Behav. Biomed. Mater..

[58] Ogurkowska M.B., Blaszezyk A. (2020). Distribution of Young’s modulus at various sampling points in a human lumbar spine vertebral body. Spine.

