# Supplementary material for: Highly Selective Interfacial Route to Eight-Functional Sucrose Methacrylate for Biocompatible Scaffold Fabrication
Source: Polymers (Basel). 2026 Jun 6;18(12):1417. doi: 10.3390/polym18121417 (PMC13306284; doi:10.3390/polym18121417)
Supplement: Supplementary file 1 [file polymers-18-01417-s001.zip › polymers-4315164-supplementary.pdf]

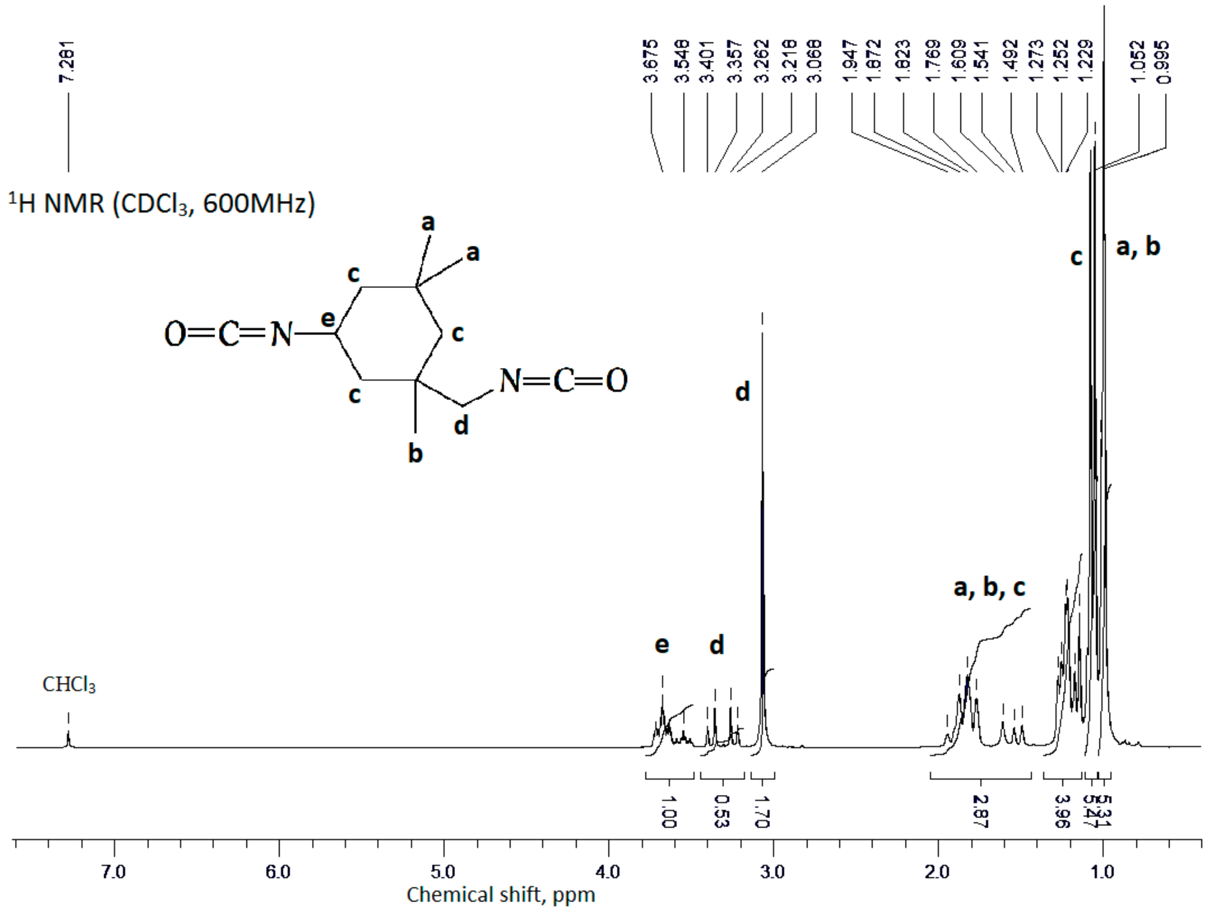

Figure S1. ¹H-NMR spectrum of the initial 3-isocyanatomethyl-3,5,5-trimethylcyclohexyl isocyanate. ¹H-NMR (($CDCl_3$) 600 MHz) δ, ppm: 0.99, 1.01 (d, 9H, CH_3 c, d), 1.05, 1.08 (d, 6H, CH_2 e), 1.09 – 1.94 (15H, CH_3 c, d, CH_2 e), 3.07 (s, 2H, CH_2 f), 3.3 (q, 2H, CH f), 3.55, 3.67 (m, 1H, CH h).

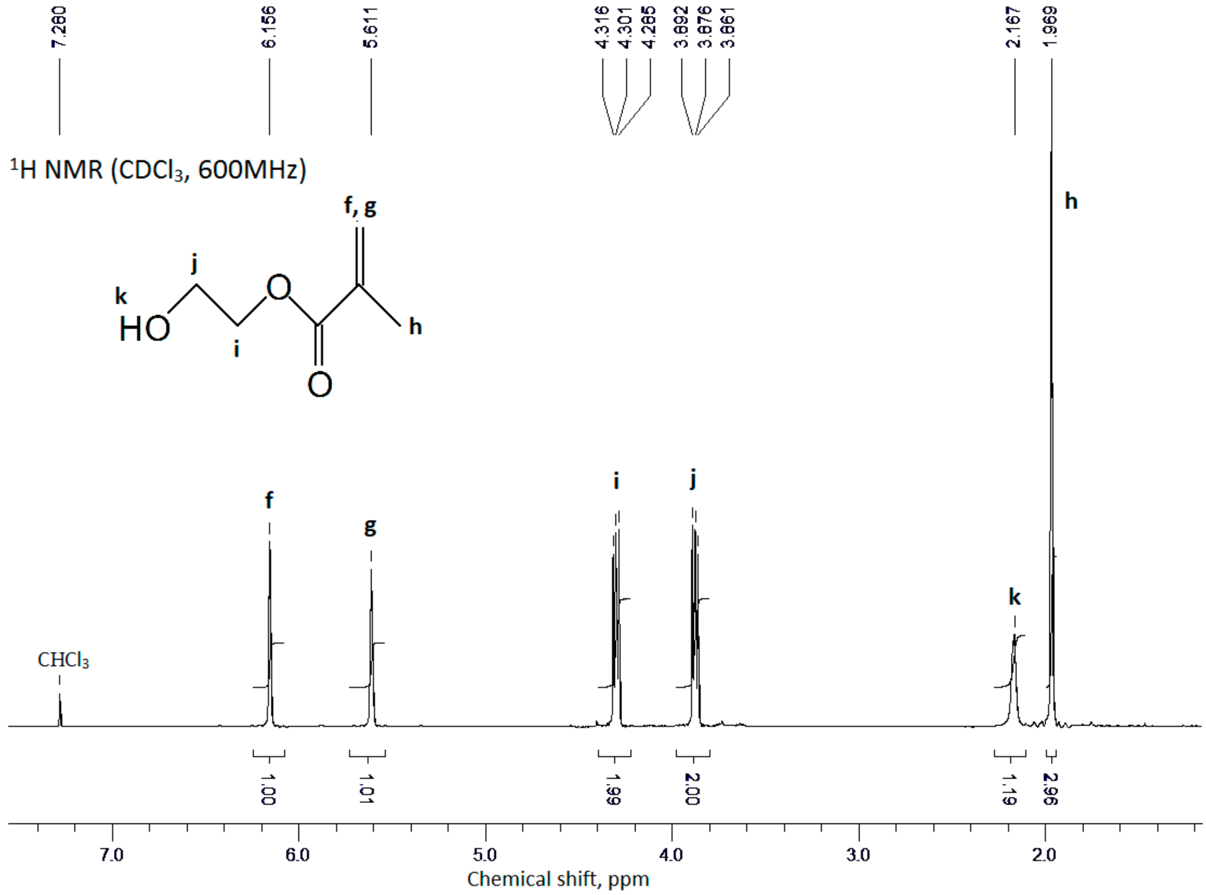

Figure S2. ^{1}H-NMR spectrum of the initial ethyleneglycol monomethacrylate. ^{1}H-NMR (($CDCl_3$) 600 MHz) δ, ppm: 1.97 (s, 3H, CH_3 n), 2.17 (s, 1H, OH p), 3.87 (t, 2H, CH_2 j), 4.30 (t, 2H, CH_2 k), 5.61 (s, 1H, CH_2 l, m), 6.15 (s, 1H, CH_2 l, m).

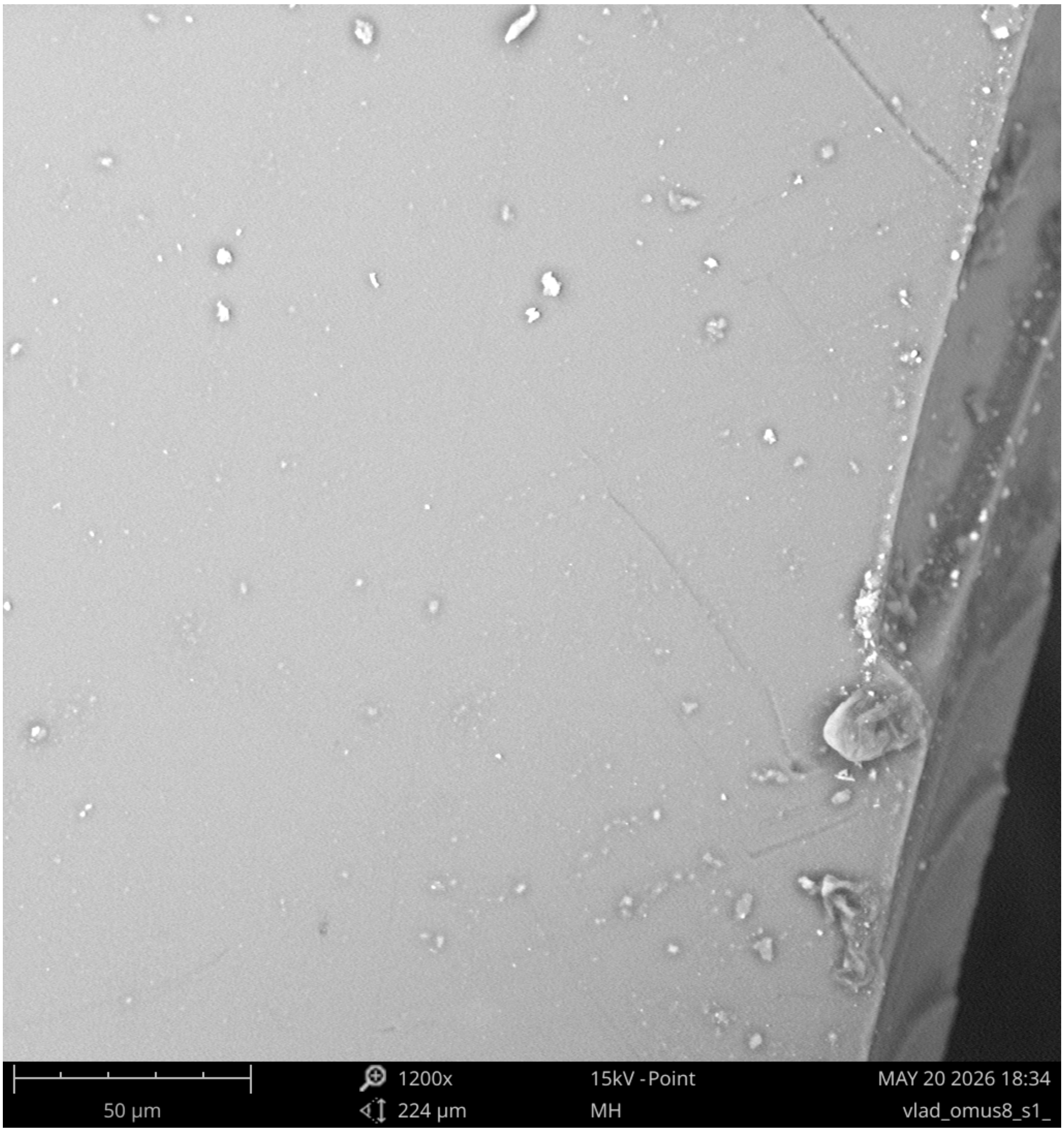

Figure S3. SEM image of the free surface and fracture surface of the UV-LED crosslinked OMUS. Magnification: x1200.

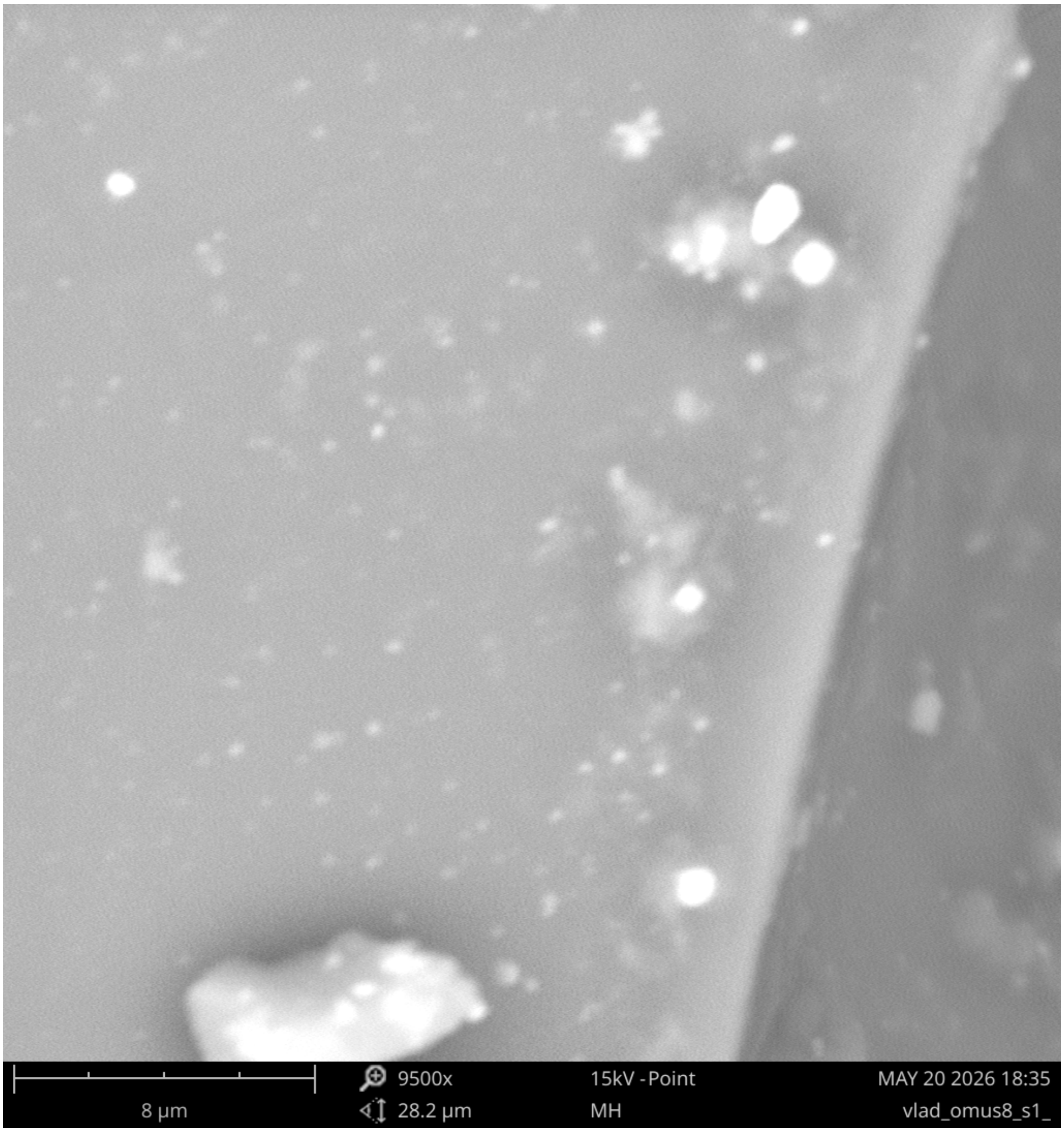

Figure S4. SEM image of the free surface and fracture surface of the UV-LED crosslinked OMUS. Magnification: x9500.
